# Usefulness of salivary alpha amylase as a biomarker of chronic stress and stress related oral mucosal changes – a pilot study

**DOI:** 10.4317/jced.51355

**Published:** 2014-04-01

**Authors:** Ravindranath Vineetha, Keerthilatha-M. Pai, Manoj Vengal, Kodyalamoole Gopalakrishna, Dinesh Narayanakurup

**Affiliations:** 1 MDS, Associate Professor. Department of Oral Medicine and Radiology, Manipal College of Dental Sciences, Manipal University. Karnataka State, India; 2 MDS, Professor and Head. Department of Oral Medicine and Radiology, Manipal College of Dental Sciences, Manipal University. Karnataka State, India; 3 MDS, Professor and Head. Department of Oral Medicine and Radiology, Vyas Dental College, Jodhpur, Rajasthan, India; 4 M.Sc, PhD. Associate professor. Department of Biochemistry, Kasturba Medical College, Manipal University, Karnataka, India; 5 MPhil, PhD, Associate Professor and Head. Department of Clinical Psychology, Kasturba Medical College, Manipal University, Karnataka, India

## Abstract

Introduction: Salivary biomarkers are suggested to provide a reliable, noninvasive and objective measurement of chronic psychosocial stress and helps in assessment of pivotal role of stress in causation or precipitation of multitude of health problems. Objectives: To evaluate the usefulness of salivary alpha amylase activity as an objective indicator of chronic stress and to find out any correlation between stress- related mucosal complaints and its levels. Study Design: Study was conducted among 50 subjects suffering from chronic stress related problems and 50 non-stressed individuals who were screened with a psychometric questionnaire. Brief case history and oral examination was carried out and about one ml of unstimulated saliva was collected. Salivary alpha amylase levels estimated were compared between study and control group and between subjects with and without oral mucosal changes using non parametric Mann Whitney U test. Results: There was statistically significant higher salivary alpha amylase levels in study group (p =.002) and salivary alpha amylase between the oral mucosal complaints group and without oral mucosal complaints group within the total study population were found to be statistically significant (p=0.045). Conclusions: Salivary amylase activity increases in patients with chronic psychosocial stress and may be used as a biomarker of chronic stress, but it may not be an indicator to suggest the development of stress related oral mucosal changes.

** Key words:**Salivary biomarker, salivary alpha amylase, psychosocial stress, sympathetic nervous system, oral mucosal changes.

## Introduction

In the modern society, stress has become an inevitable part plaguing the daily lives. Chronic psychological stress has a negative impact over physical, mental and social well-being of a person. It has been suggested to play an important role in causation or precipitation of multitude of medical and dental problems ranging from serious heart diseases, cancers, gastrointestinal diseases, to common headaches, migraine, recurrent oral ulcerations, burning and dry mouth (1-3). In order to better understand the role of stress, valid and reliable measurement of stress is of utmost importance. Since stress is a multifaceted phenomenon, it requires a multidimensional measurement approach (4). Psychometric assessment of stress component using anxiety questionnaires is highly subjective in nature but biological stress markers are suggested to provide an objective, reliable and authentic evidence of stress which is less sensitive to exaggeration. Various biomarkers used in quantification of stress include cortisol levels, immunoglobulins, chromogranin-A, cardiovascular parameters etc (4-8). Salivary bio-markers have gained wide popularity as it helps in easy, non-invasive and rapid collection of samples compared to the blood and urine samples thereby increasing the patient compliance (9-11). Few studies have provided direct evidence for the sensitivity of salivary alpha amylase levels to the changes in catecholamine levels in blood thus serving as a surrogate marker of SAM activity indicating the changes during an acute psychosocial stress (5,10,12-15). However its role in objectively assessing the chronic stress changes of body evokes more interest in the field of clinical sciences as psychosocial stress is an established risk factor for many detrimental health problems. In this study, we put an effort to evaluate the usefulness of salivary alpha amylase levels as a stress biomarker in a chronically stressed group of people and to find any correlation between these levels and commonly associated stress related oral mucosal complaints.

## Material and Methods

This study was conducted among 100 patients selected from the Departments of Clinical Psychology and Oral Medicine and Radiology. Institutional Ethics Committee approval was obtained and a detailed written informed consent was taken from each participant. Subjects were divided into Category A (study group) which consisted of 50 subjects selected from the Department of Clinical Psychology who were diagnosed to be suffering from chronic psychosocial stress after a detailed subjective and objective evaluation by the experts. Category B (control group) consisted of 50 subjects who were age and sex matched with group A, selected amongst the people visiting dental Out-patient department for routine checkup. Subjective evaluation of the anxiety levels of the individuals participating in the study was again done using a State-Trait Anxiety Inventory (STAI) questionnaire as an additional screening modality and scores were given. According to this questionnaire, score above 40 indi-cates high anxiety conditions. In our study, study group participants should also score a minimum of 40 points for both state or trait in the STAI and control group participants should score less than 40 for both state and trait anxiety questionnaires to be included in the study. The subjects in each category were subdivided into three age groups. Other exclusion criteria included those who were under 18 yrs age, smokers, pregnant, on beta blockers or any other medications, on steroid therapy in the last three months, having a history of salivary gland diseases, suffering from eating disorders (Anorexia nervosa, bulimia nervosa) or having any other medical diseases.

Detailed case history and oral examination was carried out by trained oral diagnostician to see whether the participants suffered from stress related oral mucosal -complaints such as dry mouth, burning sensation, recurrent aphthous ulcerations or lichen planus. Patients were also asked about any temporomandibular joint pain, pain in the masticatory muscles, or any atypical, vague pain in the orofacial region as these conditions may also be associated with psychological stress. Clinical diagnosis is made only if objective evidence was present at the time of oral examination and ruled out other possible differential diagnoses. Subjects were classified into group with or without oral mucosal complaints according to the presence or absence of stress related mucosal changes and findings were documented.

Saliva was collected from all the participants between 2.00 pm and 3.00 pm atleast 1hr after they were restrained from any food intake to avoid possible influence of circadian pattern and chewing activity in enzyme levels. Participants were asked to wash their mouth before saliva collection to remove any food debris. Approximately one ml of unstimulated saliva was collected by asking the patient to spit 2-3 times into a small sterile disposable plastic container and sent immediately to the clinical laboratory, Department of Biochemistry for estimation of salivary alpha amylase levels. Salivary amylase levels were estimated with Hitachi 912 Automatic analyzer using synthetic substrates. Salivary alpha amylase levels and frequency of oral findings were recorded and compared between 2 groups. Statistical analysis was done using SPSS version 15.0 Comparisons of the salivary alpha amylase values between different groups were done using non parametric Mann-Whitney U Test comparing their median and assessing the inter quartile range. Prevalence of oral mucosal lesions was compared between both groups using Chi square test and Fisher’s Exact test. p value less than/ equal to 0.05 was considered significant for each of the above mentioned analysis.

## Results

The present study to evaluate the usefulness of salivary alpha amylase as a biological marker of stress was carried out in 100 patients. Category A consisted of 50 patients who were clinically diagnosed to be under high stress or anxiety after detailed assessment by clinical psychologists (29 males and 21 females) and were sub-grouped according to their age. Category B consisted of 50 non-stressed individuals (29 males and 21 females) who were age and sex matched with category A ( Table 1), STAI scores for above 40 for all the cases of Category A (mean score-56.54) and below 40 for category B( mean score-31.66). Recurrent aphthous ulcers (RAU) were the most common mucosal complaint in the total population (34%), out of which 64.7% of RAU patients belonged to the study group. Dry mouth was observed in 56% of study group, but none in control group making this finding statistically significant. 35 participants in the study group had one or more oral mucosal complaints but only 13 participants in control group reported of oral mucosal complaints. Distribution of oral mucosal changes in study and control group is given in  Table 2.

**Table 1:**
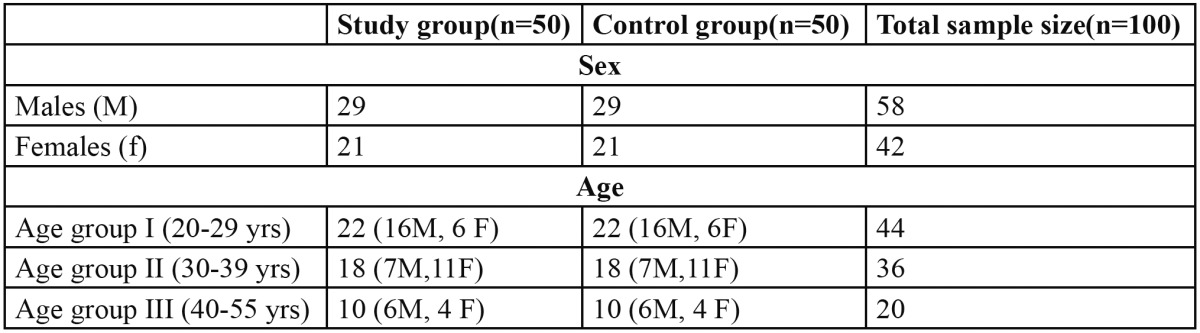
Age and gender distribution of participants in study and control group.

**Table 2:**
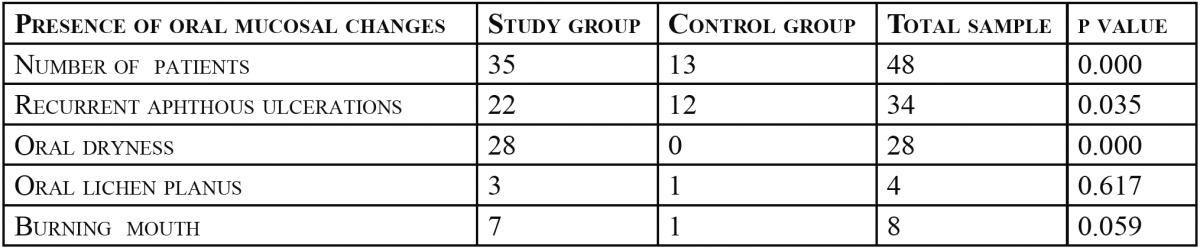
Distribution of patients with oral mucosal changes in study and control group.

**Figure 1:**
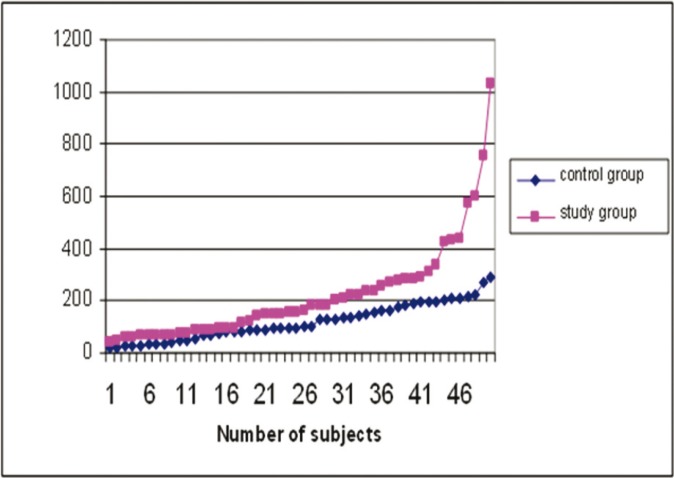
Shows the distribution of salivary alpha amylase levels between study group control group. Study group was found to have statistically significant higher values than control group (p value =.002).

Salivary alpha amylase values were compared between study group and the control group and between groups with and without any oral mucosal changes. As the data available to us was of a skewed distribution, median, rather than mean was used to compare the values between different groups. Salivary alpha amylase levels in study group showed statistically significant higher values when compared to control group (*p*=0.002) (Fig. 1). Salivary alpha amylase between the oral mucosal complaints group and without oral mucosal complaints group within the total study population were found to be statistically significant (*p*=0.045). However, the levels did not show any significant difference (*p*=0.204 in study group and *p*=0.757 within the control group) when comparison were made within the study group and control group ( Table 3).

**Table 3:**
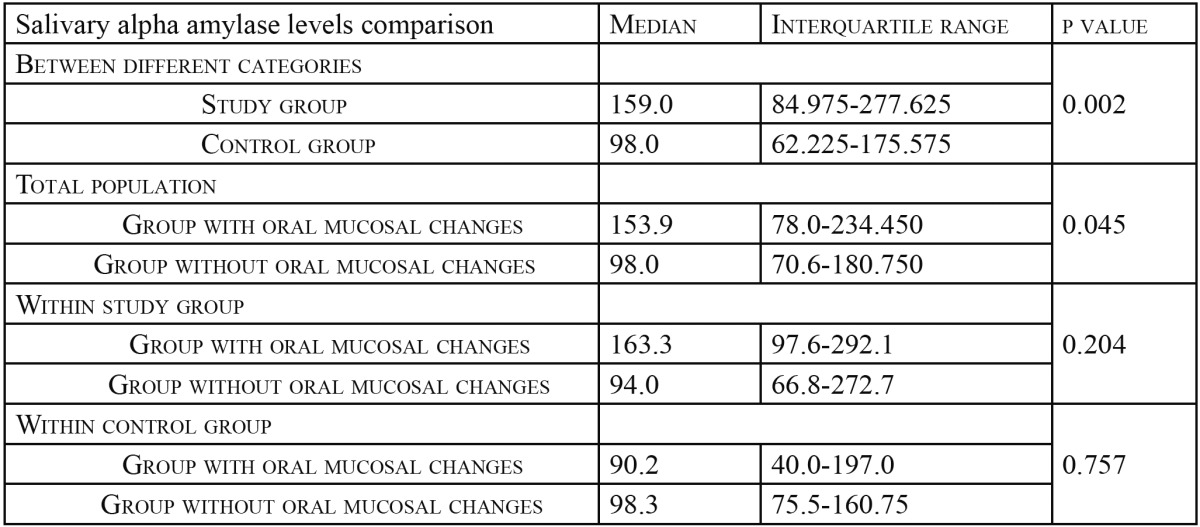
Comparison of salivary alpha amylase levels between different groups using Mann Whitney U test.

## Discussion

Role of psychosocial factors including stress in the changes of human body is one of most widely researched area of interest by psychophysiologists (5). This study was planned to evaluate the usefulness of a particular enzyme salivary alpha amylase as a biomarker of stress in chronically stressed individuals as it is said that chronic stress, rather than acute stress, usually results in extensive damage to the physical and mental well being of an individual and may cause several pathologies. Verbal or self-reporting questionnaires alone in stress evaluation provide highly inconsistent results according to patient’s mood and attitude. Many individuals suffering from stress related problems have a tendency to either deny or exaggerate the real condition; this may lead to a bias in the study and confounds with the results. This may be one of the reasons why many previous studies conducted to find out the role of psychosocial factors in the manifestations of oral pathologies have reported contradictory results.

Self reporting state trait anxiety inventory widely used to obtain a subjective assessment of stress and anxiety (6,16) was used here only as an additional screening questionnaire for the inclusion of patients to the respective groups and the scores were not correlated with salivary amylase levels in this study. McCartan BE (17) supported the view that it is more important to have overall elevated STAI scores and overall elevated biomarker levels than a direct comparison between the two. Previous study conducted by Yuka Noto *et al.* (6) found significant correlation between state anxiety scores and alpha amylase levels whereas Takai *et al.* (12) reported a significant correlation between salivary alpha amylase levels and trait anxiety scores when psychological stress was induced by stressful video viewing.

Salivary alpha amylase levels in study group showed statistically significant higher values when compared to control group (*p*=0.002). This result suggests a promising role of salivary alpha amylase as a possible biological stress marker. Increase in salivary alpha amylase during psychosocial stress may be explained on the basis of physiological response to stress. Shyuichi Shirasaki *et al.* (18) have tried to correlate salivary alpha amylase levels with pain scale of patients with chronic pain. They found a significant correlation with pain intensity and salivary alpha amylase (*p*<.01) and suggested it as a good index for measuring pain intensity. Psychosocial stress is widely known to induce various adaptational responses of physiologic systems with particular increasing activities in the hypothalamus-pituitary-adrenal axis (HPA) as well as in the sympathetic-adrenal-medullary (SAM) system. Cortisol levels reflect the HPA activity whereas salivary alpha amylase is said to reflect the SAM activity. Salivary cortisol levels were not taken into consideration in the present study as it is an established biomarker of stress reflecting the HPA activity (19-21). Many studies comparing salivary alpha amylase activity with acute stress and/ or adrenergic activity had shown that salivary alpha amylase reflected the adrenergic activity and thus might be used as a reliable index of the SAM (sympatho-adrenal medullary system) activity during stress. ( Nater *et al.* (5), Takai *et al.* (12), Bosch *et al.* (15), Ehrlert *et al.* (22). Studies done by Nater *et al.* (5), Chatterton *et al.* (23) compared both alpha amylase and cortisol levels in saliva and failed to find significant correlation. These results suggest that alpha amylase levels reflect the reaction of a different stress system than HPA axis. Takai (12) found that salivary cortisol levels showed lesser extent of increase when compared to amylase after the induction of a stressor. The latency time to peak level for cortisol was longer than that of amylase. Their results showed that psychological stressor increased the amylase levels, and the response and sensibility to the stressor were higher in amylase than those in cortisol. Schommer (24) had investigated the response of HPA and SAM activity after repeated stress and concluded that HPA responses quickly habituate, the sympathetic nervous system shows rather uniform activation patterns with repeated exposure to psychosocial challenge. The result is suggestive of utility of salivary alpha amylase in assessing chronic stress.

An increased allostatic load due to large HPA and SAM responses to repeated stress might render a subject vulnerable to various diseases; from the common cold to cardiovascular diseases in the long run. Chronic stress is also proposed to be a contributing factor in the manifestations and flare ups of several oral pathologies including lichen planus, recurrent aphthous ulcerations, burning mouth, atypical facial pain and xerostomia (25-32). We observed the prevalence of oral mucosal abnormalities with a suggested stress correlation. 48% of the population had oral mucosal changes, out of which maximum patients belonged to study group. Oral mucosal changes/ abnormalities frequently encountered were recurrent aphthous ulcers, lichen planus, burning sensation and dry mouth. 70% of the subjects in the study group had one or more oral complaints that may have association with stress while only 26% in the control group had such complaints. Common complaint as well as finding observed in the study group was oral dryness followed by recurrent aphthous ulcers. Prevalence of RAU in study group was statistically significant when compared to the control group (*p*=0.035).This is in concordance with many other studies, which suggested association of RAU with stress (17,27). Many studies in the past have tried to find out the association of stress and oral lesions, but none, to the best of our knowledge, showed the prevalence of different oral lesions in chronically stressed individuals. Our sample size was less to represent the prevalence of stress- related changes in the general population. Previous studies to find out the correlation between lichen planus and stress reported conflicting results. Burkhart et al. (28), Hampf *et al.* (30), and Rojo-Moreno *et al.* (33) found significant association of stress and anxiety with oral lichen planus whereas Macleod (34), Allen et al. (35) found no significant association of stress and anxiety with lichen planus. Lichen planus and burning mouth were less frequent findings (4% and 8% respectively) in total population and there were no statistically significant difference between control and study group. Our study suggests the lesser prevalence of these conditions among the selected population. Burning mouth syndrome, atypical facial pain, or masticatory muscle pain were not reported in our population although all these conditions are suggested to be associated with psychological factors.

The results of comparison of salivary alpha amylase levels between groups with and without oral mucosal changes suggest that salivary alpha amylase levels do not have a correlation with oral mucosal changes observed in the study. A subject with higher salivary alpha amylase does not have the greater tendency towards developing stress related oral mucosal changes.

Our study had to deal with possible constraints. Reliability of STAI scores alone in selection of control population is questionable, but we hypothesized that the psychological stress in patients seeking treatment for their stress related problems would be very much higher than those who can cope up with their stress themselves. The severity of stress suffered by each patient in study group was not considered in the study. Whether higher levels of alpha amylase in these patients was a reflection of their chronic stress status or was due to the acute exacerbation could not be differentiated. Even though, the sample size was statistically sufficient, study using a larger sample size is recommended to confirm our findings. Lastly, not many studies have been reported in the literature, which could provide more information about the usefulness of salivary alpha amylase in chronic psychological stress. Further research in this aspect in comparison with various biomarkers is warranted to establish our findings.

## Conclusion

In our study, we found that salivary alpha amylase activity increases in patients with chronic psychosocial stress and may be used as a biomarker of chronic stress, but it may not be an indicator to suggest the development of stress related oral mucosal changes. Dry mouth and recurrent aphthous ulcerations were the most common oral mucosal changes seen in chronically stressed individuals but further studies involving a large study population are required to substantiate our results. This is a preliminary study, limited by its sample size, but the design, findings, and inclusion of physiological measures present a contributory role in the essential line of research.
